# QIP: Liaison Psychiatry Outcome Measures at University College Hospital in London

**DOI:** 10.1192/bjo.2022.273

**Published:** 2022-06-20

**Authors:** Ariana Auyeung, Lisa Patel

**Affiliations:** 1 University College London Hospital, London, United Kingdom; 2 Camden and Islington NHS Foundation Trust, London, United Kingdom

## Abstract

**Aims:**

The purpose of this quality improvement project was to improve the collection of outcomes in the Liaison Psychiatry (LP) department at the University College Hospital in London (UCLH). To achieve this, the Framework for Routine Outcome Measurement in Liaison Psychiatry (FROM-LP) was used to gather data and evidence on clinical and other patient-related outcomes provided by the department. The FROM-LP was created to provide a consistent way to compare the quality and performance of Liaison Psychiatry services across the NHS. It was developed in 2015 and is based on the most widely used measurement frameworks for assessing quality and performance of services.

**Methods:**

This project implemented the FROM-LP, using the Identify and Rate the Aim of the Contact (IRAC) tool and the Clinical Global Impression – Improvement scale (CGI-I) from September to November 2021 in the UCLH Liaison Psychiatry department. The PDSA (plan, do, study, and act) cycle was used to carry out this quality improvement project and the data were collected by two foundation year doctors.

The IRAC scale identified ten categories for the aim of contact by LP and a rating on whether the aim was fully achieved, partially achieved, or not achieved after patient contact. The CGI-I scale was used to rate whether a patient had improved upon discharge by LP. Data were also collected on the demographics of patients, the specialty teams that referred to LP, whether legal frameworks were used, and where patients were discharged to.

**Results:**

This project improved the collection of outcome data in the department from 0% to 98.16%, indicating an improvement of outcomes measurement by >98%. Other outcomes collected showed that patients were predominantly 21–30 years of age and referred to community mental health teams when discharged. The IRAC tool showed most patients were referred for assessment and diagnosis, with the majority of these aims marked as ‘fully achieved’. The CGI-I tool showed most patients were ‘much improved’ upon discharge.

**Conclusion:**

The collection of these outcomes led to the creation of an outcomes measure form on the primary electronic software system (Carenotes) utilized by the department and local trust. This electronic form is now currently being used by the Liaison Psychiatry department at UCLH for their patients and makes this improvement sustainable while providing an easier means to continue collecting data. Ultimately, the collection of these outcomes will guide future changes and improvements for both the liaison psychiatry department and its patients.

